# Requirement of the immediate early gene *vesl-1S/homer-1a *for fear memory formation

**DOI:** 10.1186/1756-6606-2-7

**Published:** 2009-03-05

**Authors:** Naoko Inoue, Harumi Nakao, Rika Migishima, Toshiaki Hino, Minoru Matsui, Fumihiko Hayashi, Kazuki Nakao, Toshiya Manabe, Atsu Aiba, Kaoru Inokuchi

**Affiliations:** 1 Mitsubishi Kagaku Institute of Life Sciences, MITILS, 11 Minamiooya, Machida, Tokyo 194-8511, Japan; 2 Department of Mental Retardation and Birth Defect Research, National Institute of Neuroscience, Tokyo 187-8502, Japan; 3 Division of Molecular Genetics, Department of Physiology and Cell Biology, Kobe University Graduate School of Medicine, Kobe 650-0017, Japan; 4 Institute of Medical Science, University of Tokyo, Tokyo 108-8639, Japan; 5 Department of Pharmacy, Chiba Institute of Science, Choshi, Chiba 288-0025, Japan; 6 Laboratory for Animal Resources and Genetic Engineering, RIKEN Center for Developmental Biology, Kobe 650-0047, Japan; 7 Japan Science and Technology Agency, CREST, Kawaguchi 332-0012, Japan

## Abstract

**Background:**

The formation of long-term memory (LTM) and the late phase of long-term potentiation (L-LTP) depend on macromolecule synthesis, translation, and transcription in neurons. *vesl-1S *(*V*ASP/*E*na-related gene upregulated during *s*eizure and *L*TP, also known as *homer-1a*) is an LTP-induced immediate early gene. The short form of Vesl (Vesl-1S) is an alternatively spliced isoform of the *vesl-1 *gene, which also encodes the long form of the Vesl protein (Vesl-1L). Vesl-1L is a postsynaptic scaffolding protein that binds to and modulates the metabotropic glutamate receptor 1/5 (mGluR1/5), the IP_3 _receptor, and the ryanodine receptor. Vesl-1 null mutant mice show abnormal behavior, which includes anxiety- and depression-related behaviors, and an increase in cocaine-induced locomotion; however, the function of the short form of Vesl in behavior is poorly understood because of the lack of short-form-specific knockout mice.

**Results:**

In this study, we generated short-form-specific gene targeting (KO) mice by knocking in part of *vesl-1L*/*homer-1c *cDNA. Homozygous KO mice exhibited normal spine number and morphology. Using the contextual fear conditioning test, we demonstrated that memory acquisition and short-term memory were normal in homozygous KO mice. In contrast, these mice showed impairment in fear memory consolidation. Furthermore, the process from recent to remote memory was affected in homozygous KO mice. Interestingly, reactivation of previously consolidated fear memory attenuated the conditioning-induced freezing response in homozygous KO mice, which suggests that the short form plays a role in fear memory reconsolidation. General activity, emotional performance, and sensitivity to electrofootshock were normal in homozygous KO mice.

**Conclusion:**

These results indicate that the short form of the Vesl family of proteins plays a role in multiple steps of long-term, but not short-term, fear memory formation.

## Background

Memory has at least two distinct forms, which include short-term and long-term memory, the latter of which lasts days, weeks, or years. Formation of long-term memory (LTM), but not short-term memory (STM), requires the synthesis of new RNA and protein [1]. In order to understand the molecular basis of memory storage, it is important to identify and characterize genes whose expression is altered during memory formation. Many studies have been carried out to identify neural activity-regulated genes potentially involved in the formation of LTM [2-9]. One of the candidates thus isolated is the *vesl-1S/homer-1a *gene [4,10].

**Figure 1:**
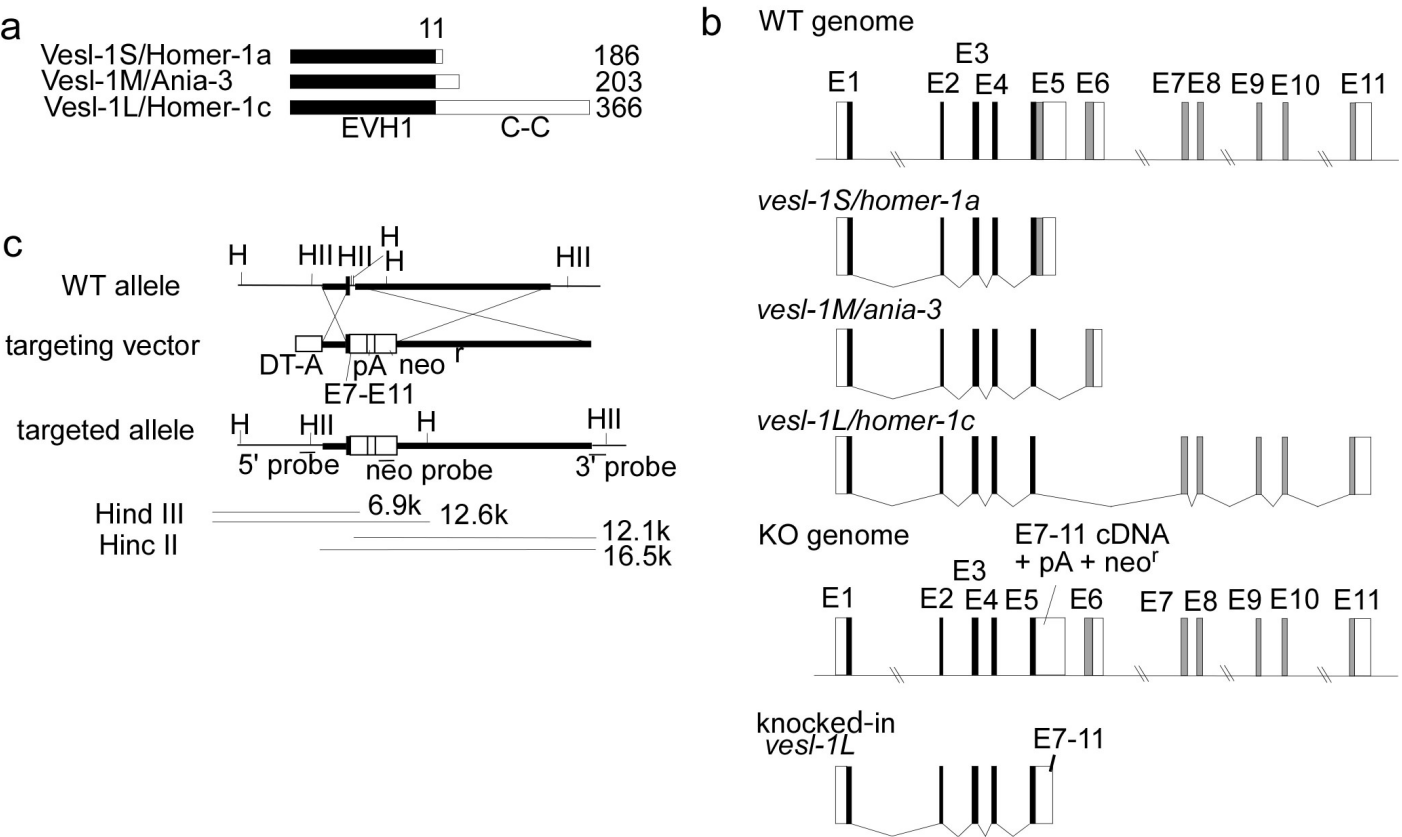
**Targeting construct**. *a*, Protein structure of Vesl-1S (186 amino acids) and Vesl-1L (366 amino acids). The 175 N-terminal amino acids are shared (black box). White boxes represent sequences unique to each isoform (11 (Vesl-1S) and 191 (Vesl-1L) amino acids). EVH1; Ena-Vasp homology domain 1. *b*, Genomic structure of the *vesl-1 *gene family. Gray boxes represent genomic regions specific to each isoform. White boxes represent the 5' and 3' untranslated regions of each isoform. Black boxes represent the regions shared among the three isoform [23,24]. The genomic structure of KO mice are also shown for comparison (bottom). Figure 1b is not in scale. E1-E11, exon 1–11. *c*, Targeting construct. The thick bar on the left represents the 3' region of intron 4 and the following vertical thick bar represents the exon 5 shared region. The thick bar on the right represents intron 5, downstream of the *vesl-1S*-specific region. Crossed lines denote the homologous recombination site. pA, SV40 poly A sequence. neo^r^, neomycine resistant gene including promoter and bovine growth hormone poly(A). DT-A, diphtheria toxin A fragment.

The *vesl-1 *gene encodes three isoforms, which include Vesl-1S/Homer-1a, Vesl-1M/Ania-3, and Vesl-1L/Homer-1c, in mice (Figure 1) [11,12]. The *vesl-1S *and *vesl-1M *mRNAs (short forms) genes are upregulated as immediate early genes during convulsive seizures and LTP [4,10]. In contrast, the *vesl-1L *mRNA (long form) is constitutively expressed. These proteins bind via their common N-terminal EVH-1 domain to group 1 metabotropic glutamate receptors (mGluRs), inositol tri-phosphate receptors (IP_3_Rs), ryanodine receptors (RyRs), the Shank family of postsynaptic scaffold proteins, and the C-type transient receptor potential channel (TRPC) [11-17]. Vesl-1L/Homer1c contains additional C-terminal sequences, which include a coiled-coil domain and a leucine zipper, via which they associate to form homo- and heteromultimers to function as a scaffold protein and interact with receptors [11,12,14]. Although the short forms of the Vesl protein contain the EVH-1 domain, they lack the C-terminal coiled-coil domain; therefore, the inducible short form Vesl-1 proteins are believed to act as endogenous dominant-negative regulators, as they compete with the long form Vesl protein to bind to receptors and channels.

Studies using *vesl-1 *and *vesl-2 *null mutant mice reveal important roles for this family of proteins in addiction [18,19], schizophrenia [20], and ethanol sensitivity [21]. Vesl-1S overexpression by *in vivo *AAV virus infection in rat hippocampus leads to impaired ability in the water maze test [22]. These observations strongly suggest that the Vesl-1 proteins play a role in the regulation of physiological functions and pathological behavior *in vivo*, via the modulation of glutamatergic transmission; however, the function of the short form of Vesl in memory formation is not well understood.

In this study, we used gene targeting techniques to generate short-form-specific KO mice, to clarify the role of the short form of Vesl-1 in LTM formation. The analysis of several behavioral paradigms demonstrated that specific KO of the short form affects several processes of fear memory formation.

## Results

### Generation and characterization of *vesl-1 *short-form-specific KO mice

The 175-amino-acids N-terminal sequence is shared by the Vesl-1S, Vesl-1M, and Vesl-1L proteins, whereas the C-terminal 11 amino acid sequence is specific to the Vesl-1S protein (Figure 1a) [23,24]. To generate short-form-specific gene-targeting mice, genomic DNA corresponding to the C-terminal 11 amino acids was replaced by cDNA encoding Vesl-1L-specific C-terminal amino acids (knock in of exons 7–11) (Figure 1b and Figure 1c). These mice were expected to produce Vesl-1L protein, but not Vesl-1S and Vesl-1M proteins.

**Figure 2:**
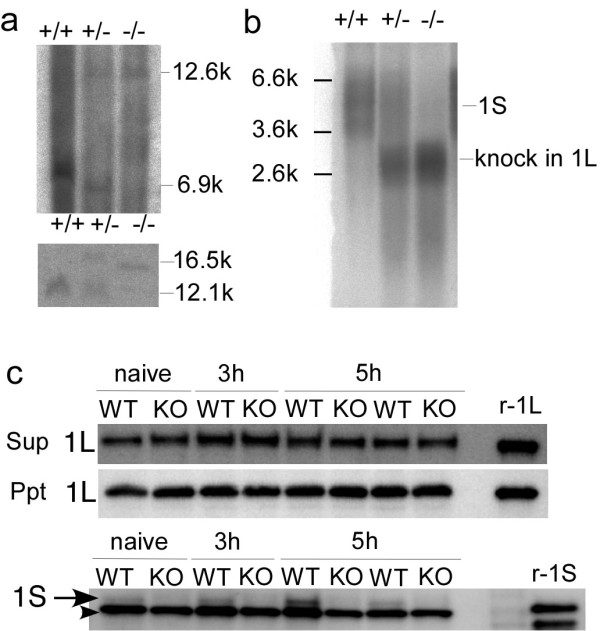
**Generation of short-form-specific KO mice**. *a*, Southern blotting. HindIII (H) digestion was probed using a 5' probe (upper panel). HincII (HII) digestion was probed using a 3' probe (lower panel). *b*, Northern blotting. *c*, Western blotting. Mice were subjected to MECS and were sampled 3 or 5 h later (see Materials and Methods for details). An anti-Vesl-1L antibody (upper panels) or anti-Vesl-1S antibody (lower panel) was used for immunoblotting. Anti-Vesl-1L antibody recognized both Vesl-1L and Vesl-1L-Δ12. Arrow, Vesl-1S; arrowhead, non-specific signal that was detected by antibody we used; r-1L, recombinant Vesl-1L; r-1S, recombinant Vesl-1S.

Heterozygous offspring were intercrossed to produce homozygous mutants, and the offspring were screened for homologous recombination by Southern blot analysis. Wild-type (WT) and mutant alleles were identified by the presence of 6.9 kb and 12.6 kb HindIII-HindIII fragments for the 5' probe, and the presence of 12.1 kb and 16.5 kb HincII-HincII fragments for the 3' probe, respectively (Figure 2a). Northern blot analysis showed that homozygous mice expressed the knocked-in *vesl-1L *transcripts, but not the endogenous *vesl-1S *transcripts (Figure 2b). Henceforth, homozygous mice are referred to as KO mice.

The number of KO mice in the offspring followed a Mendelian inheritance (male: wild type, 187 (1.00); heterozygous, 388 (2.07); KO, 182 (0.97); female: wild type, 193 (1.00); heterozygous, 371 (1.92); KO, 182 (0.94)). KO mice appeared healthy and mated normally, and their overall behavior was indistinguishable from that of wild-type and heterozygous littermate mice.

**Figure 3:**
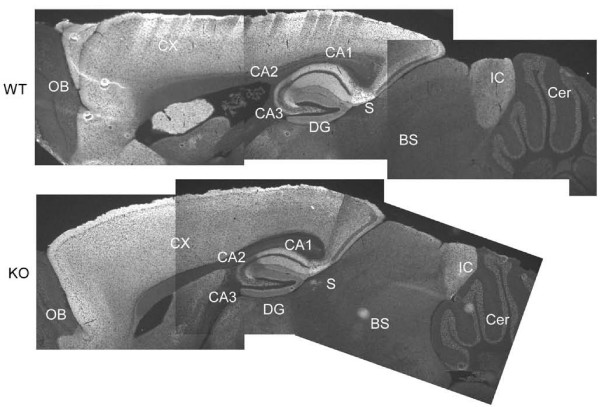
**Immunohistochemistry using a Vesl-1L-specific antibody**. Mice were subjected to MECS and brains were dissected 5 h later. A sagital section is shown. IC, inferior colliculus; OB, olfactory bulb; S, subiculum; Cx, neocortex; BS, brain stem; Cer, cerebellum.

Neural activity-dependent regulation of alternative RNA splicing is mediated by activity-dependent selection of poly(A) sites that are located at the 3' end of exons 5 and 6 [23,24]. Because genomic DNA of KO mice lacked these poly(A) sites and the expression of knocked-in *vesl-1L *mRNA was directed by the endogenous *vesl-1 *promoter, we wondered whether the level of Vesl-1L protein was augmented in a neural activity-dependent manner. Western blot analysis revealed that the level of Vesl-1L protein did not increase after the application of a maximum electroconvulsive shock (MECS) in the hippocampus of KO mice and was comparable to that of wild type mice in the condition where the level of Vesl-1S protein was increased (Figure 2c). Furthermore, the region specificity of the Vesl-1L protein was conserved between wild-type and KO mice (Figure 3). Regions that expressed Vesl-1L protein at high levels included the cortex, the CA1, the CA3, and the dentate gyrus of the hippocampus, the subiculum, and the inferior colliculus. Low Vesl-1 expression levels were detected in the CA2 area of the hippocampus, the olfactory bulb, the brain stem, and the cerebellum; thus, we successfully generated short-form-specific KO mice in which the expression of Vesl-1L protein was unaltered.

### KO mice showed rough dendritic appearance in MECS-treated tissue

**Figure 4:**
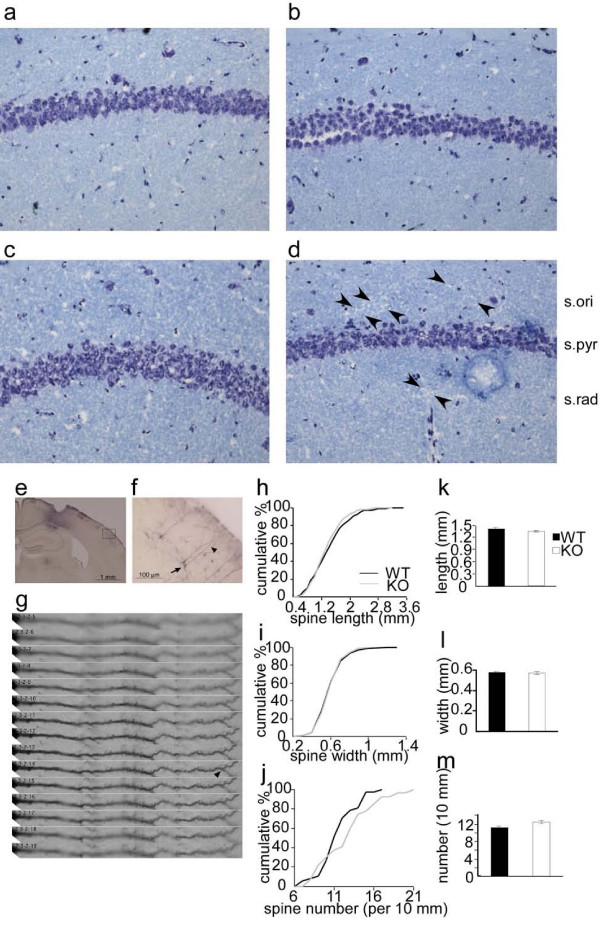
**Normal spine morphology in KO mice**. *a-d*, Histopathological analysis of hippocampus using thionine staining. *a*, naive WT mice; *b*, WT mice after MECS (3 h); *c*, naive KO mice; *d*, KO mice after MECS (3 h). Arrowheads, sparse and damaged area. s. ori, sriatum oriens; s. pyr, striatum pyramidale; s. rad, striatum radiatum. *e-m*, A biotinylated tracer was injected into the dorsal hippocampus. Pyramidal cells in cortical layer II-III were visualized using avidin and DAB. *e-g*, Examples of neuronal morphology. e, Low-magnification image. f, Magnified image of boxed region in (e). g, High-magnification images of the first dendritic branch of pyramidal neurons surrounded by an arrow and an arrowhead (the first branch point) in (f). Serial images at 50 nm interval. Cumulative frequency of spine length (*h*), spine width (*i*), and spine number (*j*). *k*, The average of spine length. WT, 594 spines from 5 neurons, 6–7 dendrites per neuron; KO, 501 spines from 4 neurons, 5–7 dendrites per neuron; *P *= 0.09, *t *test. *l*, Spine width. WT, 594 spines from 5 neurons, 6–7 dendrites per neuron; KO, 502 spines from 4 neurons, 5–7 dendrites per neuron; *P *= 0.49, *t *test. *m*, Number of spines. WT, 37 dendrites from 5 neurons; KO, 27 branches from 4 neurons; *P *= 0.12, *t *test. Scale bar, 1 mm (*e*), 100 μm (*f*).

Adult brains were examined by thionine staining to assess possible histopathological abnormalities (Figure 4). The overall structure of the brain was normal in KO mice (Figure 3). In the hippocampal CA1 region, however, KO mice showed a mildly rough and sparse dendritic layer in the naive condition (Figure 4c) and a more severely damaged dendritic layer 3 h after MECS (Figure 4d). On the other hand, wild type mice showed normal appearance of dendritic layer both in naive condition (Figure 4a) and 3 h after MECS treatment (Figure 4b); thus, regarding the pathology of the dendritic layer, KO mice were more vulnerable to damage caused by MECS.

### Spine number and morphology were normal in KO mice

The Vesl family of proteins is suggested to be involved in synaptogenesis [4], regulation of the actin cytoskeleton [25,26], and regulation of synaptic morphology [27-29]. We examined the number and the morphology of spines in the somatosensory and auditory cortex in naive adult mice *in vivo *(Figure 4e, f, g, h, i, j, k, l, m). The average spine length (Figure 4h, k) and width (Figure 4i, l), and the number of spines on the first branch of dendrites (Figure 4j, m), were similar between wild-type and KO mice.

### Memory acquisition and STM were normal in KO mice in contextual fear conditioning

**Figure 5:**
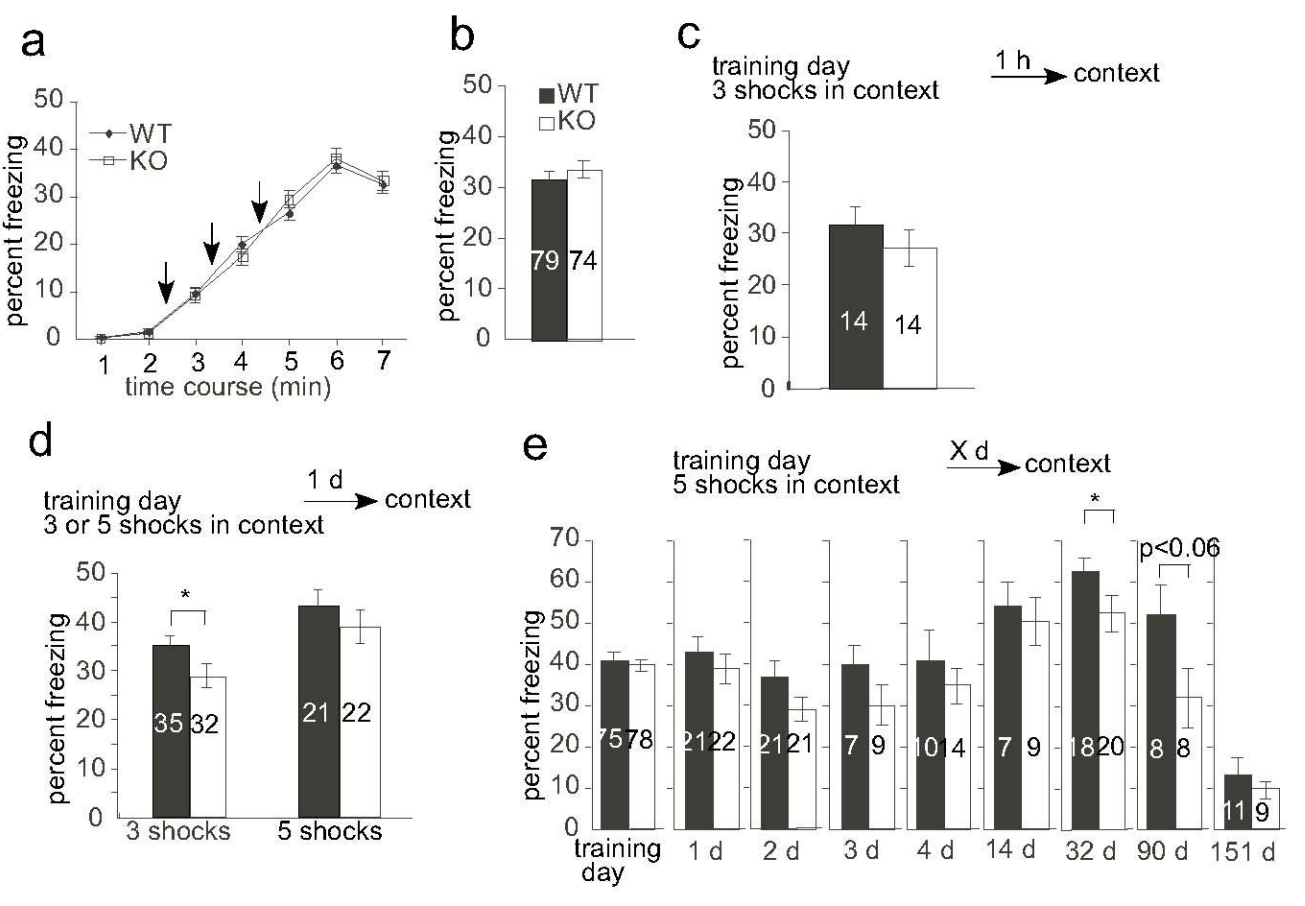
**KO mice showed normal STM and a defect in LTM and in the process from recent to remote memory**. *a*, Acquisition of freezing responses during the training trial. Three footshocks (arrows) were administered in a conditioning chamber. The mean percentage of time spent in freezing is plotted per 1 min. *b*, Average freezing during the last 3 min of the training session (*P *= 0.43, *t *test). *c*, Experimental design and conditioned freezing responses 1 h after the training. The mean percentage of time spent in freezing during the 6 min test session is plotted (*P *= 0.39, *t *test). *d*, Experimental design and conditioned freezing responses 24 h after the training. During the training session, mice received either 3 or 5 footshocks. *e*, Experimental design and conditioned freezing responses. Mice received 5 footshocks and freezing responses were assessed for 6 min at the test day indicated. No significant differences in freezing responses were detected, with the exception of the 32-day test, in which KO animals showed a decreased freezing response when compared with WT mice. The number of mice used for each experiment is indicated in the graph. Note that different groups of mice were used for each experiment. *, *P *< 0.05, *t *test.

We assessed learning and memory using the contextual fear conditioning that is dependent on the hippocampus and amygdala [30]. Both wild type and KO mice showed a similar increase in the freezing behavior during conditioning under three footshock conditions (Figure 5a, b). A measurement of freezing response 1 h after the conditioning session showed that KO mice exhibited normal freezing response when compared with the wild type littermates (Figure 5c); thus, KO mice showed normal acquisition and STM during contextual fear conditioning.

### KO mice showed impairment in fear memory consolidation, which was rescued by intense training

Mice were conditioned by administration of three shocks and were then returned to their homecage. Twenty-four hours later, mice were again placed in the conditioning chamber: although memory acquisition was comparable between KO and wild type mice, as described in the previous section, KO mice showed less freezing response than wild-type mice in this test (wild-type, 35.3%; KO, 28.9%; *P *< 0.05, *t *test) (Figure 5d). In contrast, KO mice conditioned by application of five shocks showed an equivalent level of freezing to that of wild type animals (wild-type, 43.2%; KO, 39.0%; *P *= 0.38) (Figure 5d); thus, KO mice exhibited a defect in fear memory consolidation, which was rescued by robust acquisition. This suggests a mild impairment in the consolidation process.

### Memory retention was affected in KO animals

Mice were conditioned by the application of five shocks and were returned to the conditioning chamber at varying intervals, using a separate group of mice for each condition. Both genotypes of mice displayed a similar degree of conditioned freezing to the context, up to 14 days. After 32 days, however, KO mice showed significantly less freezing than wild-type mice (wild-type, 62.8%; KO, 52.2%; *P *< 0.05, *t *test) (Figure 5e). This suggests that the long-lasting memory retention was impaired in KO mice.

### Reactivation attenuated the freezing response in KO mice

**Figure 6:**
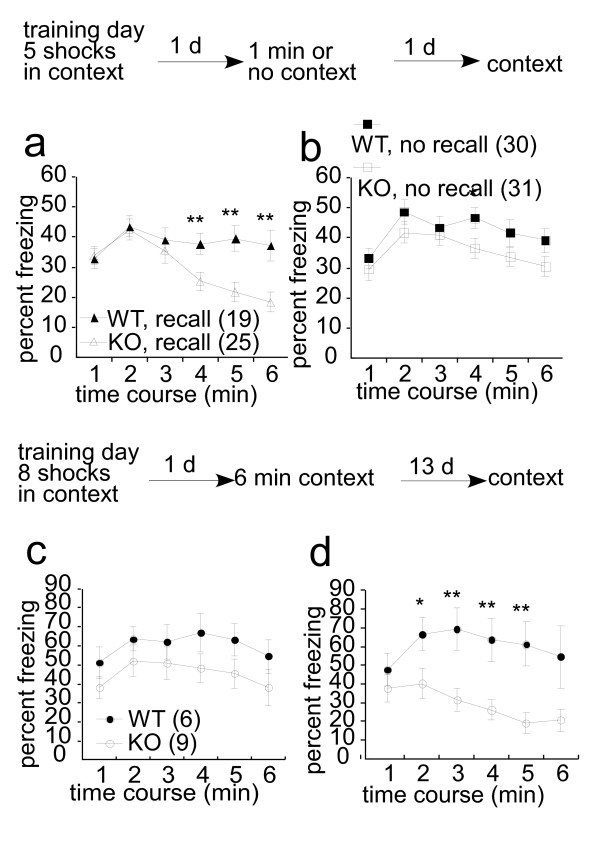
**Reactivated memory becomes labile in KO mice**. At the training day, mice received 5 (*a, b*) or 8 (*c, d*) footshocks. *a-b*, The mean percentage of time spent in freezing 2 d after the training is plotted per 1 min. *a*, 1 min context at day 1. *b*, No recall. *c-d*, The mean percentage of time spent in freezing 1 d (*c*) or 14 d (*d*) after the training is plotted per 1 min. The number of mice used for each experiment is indicated in parentheses. A *t *test was performed between KO and WT at each time point; *, *P *< 0.05; **, *P *< 0.01; *t *test.

Memory retrieval, which induces a state of plasticity in which memories become labile that is followed by stabilization [31,32]. The stabilization of memory after retrieval is called reconsolidation. The molecular mechanisms underlying memory consolidation and reconsolidation are partially shared [32-35]. We next assessed whether reconsolidation of the retrieved memory depended on the short form of Vesl-1 (Figure 6). Mice were fear conditioned to the context by five shocks. Twenty-four hours later, one group of mice was reexposed to the training context for 1 min without footshock and freezing response was assessed the following day. Both wild type and KO mice showed an equal level of freezing during the 1-min reexposure to the training context (data not shown). This 1-min reactivation promoted a decrease in freezing response in KO mice when compared with wild type mice (*P *< 0.05, repeated measures ANOVA) (Figure 6a). A separate group of mice were treated essentially as described above, with the exception that the 1-min of context reexposure was omitted. During the subsequent test, wild-type and KO mice showed a similar level of freezing (*P *= 0.13, repeated measures ANOVA) (Figure 6b); thus, the reexposure to the training context was critical to the amnesic effects of the deletion of the short form of Vesl-1 following memory reactivation.

A similar result was obtained from a different experimental schedule (Figure 6c, d). Mice received eight footshocks during the training day. Twenty-four hours later, mice were re-exposed to the training context for 6 min without footshock and were tested 13 days later. Freezing behavior during the 6-min reexposure was not significantly different between wild type and KO mice (Figure 6c); however, KO mice showed significantly less freezing when tested 13 days later (Figure 6d, *P *< 0.01, repeated measures ANOVA). Fear memory at day 14 under the five shock conditioning was normal in KO mice (see Figure 5e), which suggests that fear memory at day 14 under the eight footshock condition was also normal in KO mice. We suggest that reexposure to context at day 2 results in a decrease in freezing behavior in KO mice at day 14. Taken together, these results suggest that KO mice have an impaired reconsolidation process.

### Within-session freezing decrement was faster in KO mice

**Figure 7:**
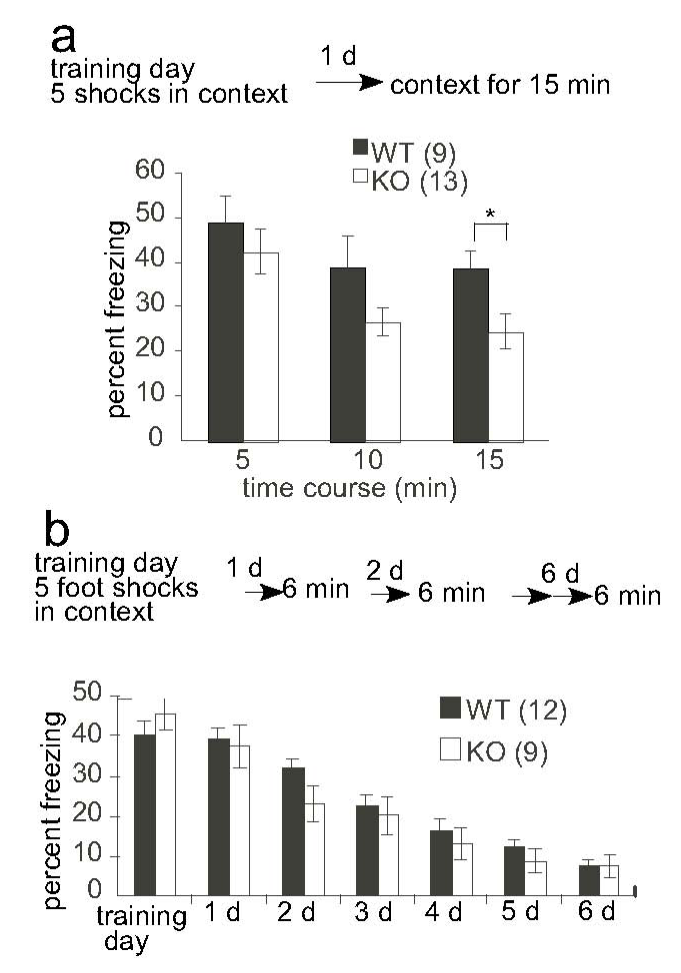
**KO mice show faster within-session freezing decrement**. *a*, Mice were conditioned by administration of 5 footshocks and the freezing response was measured after 24 h without footshock for 15 min. *, *P *< 0.05, *t *test. *b*, KO mice showed normal freezing decrement in the consolidation of extinction. Mice were conditioned by 5 footshocks. Mice were exposed to the conditioning chamber without footshock every day to assess freezing response. The mean percentage of time spent in freezing during the 6 min test is plotted. The number of mice used for each experiment is indicated in parentheses.

Fear memory retrieval without unconditioned stimulus initiates two opposite processes: reconsolidation and extinction [31,32,36]. Reconsolidation acts to stabilize memory, while extinction tends to weaken the expression of original memory. We next determined whether deletion of the short form of Vesl-1 affected the extinction of fear-conditioned memory. Mice received five footshocks during training day and freezing behavior was assessed 24 h later via 15-min exposure to the context (Figure 7a). KO mice showed faster within-session freezing decrement.

### Memory extinction was normal in KO mice

Figure 7b shows the results of both learning and formation of memory extinction. Mice received five footshocks during the training day. Formation of extinction was measured by daily recall for 6 min without footshock. Repeated exposure to the context without footshock decreased freezing response, whereas no difference was observed in inter-session freezing decrement between wild-type and KO mice (*P *= 0.42, repeated measures ANOVA).

### General activity and emotional performance were normal in KO mice

**Figure 8:**
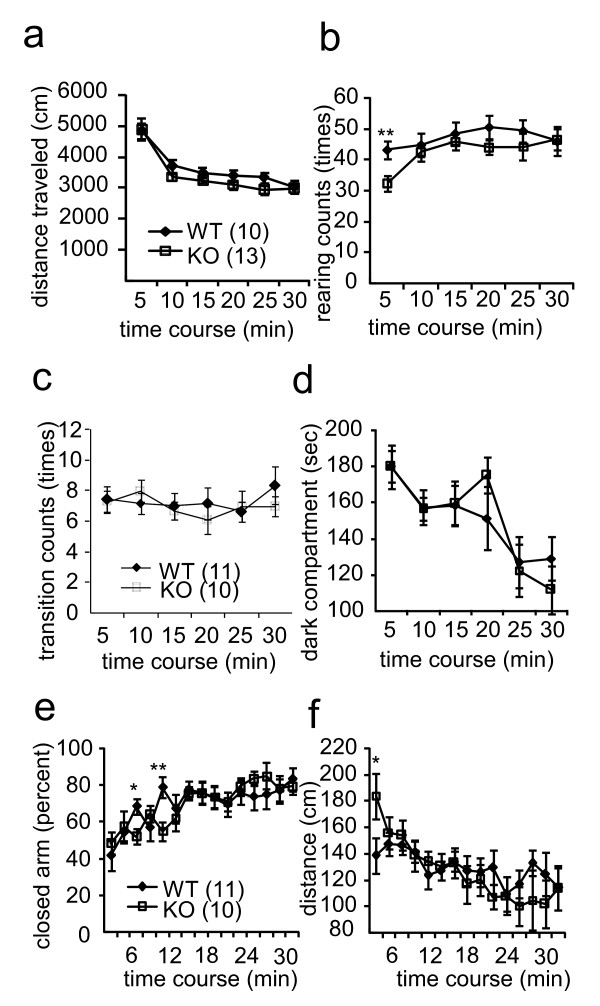
**General locomotor activity and emotional performance were normal in KO mice**. *a-b*, Open-field test. The means of locomotion distance (*a*) and number of rearings (*b*) are presented for each block of 5 min. *c-d*, Light-dark choice test. *c*, Number of transitions from the light to the dark compartment. *d*, Stay time in the dark compartment. *e-f*, Elevated plus-maze test. *e*, Stay percent in the closed arm. *f*, Traveling distance in the closed arm. The number of mice used for each experiment is indicated in parentheses. **, *P *< 0.01; *, *P *< 0.05; *t *test.

General activity and emotional performance were assessed using the open-field test, the light-dark test, and the elevated plus-maze test (Figure 8). The general activity test revealed that locomotor distance and the number of rearings were not significantly different between wild-type and KO mice (Figure 8a, b). The light-dark test showed that KO mice exhibited a behavior that was comparable to wild-type mice (Figure 8c, d), in which no significant differences were observed in the number of transitions from the light to the dark compartment and in the length of stay in the dark compartment. The elevated plus-maze test revealed that both stay time and traveling distance in the closed arm were similar between wild-type and KO mice (Figure 8e, f).

**Figure 9:**
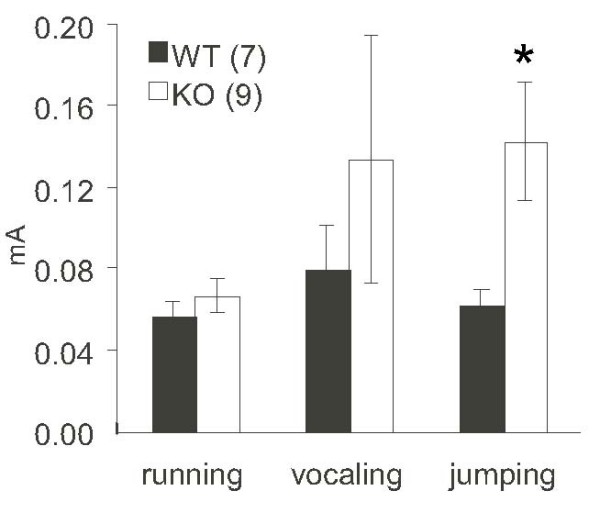
**Sensitivity to electrofootshock**. Vertical values indicate the minimal level of footshock current required to elicit three stereotypical responses: running, vocalization, or jumping. The number of mice used for each experiment is indicated in parentheses. *, *P *< 0.05; *t *test.

Nociceptive reactions to footshock were also evaluated (Figure 9). We determined the level of current required to elicit three stereotypical responses in mice. There were no significant differences in the minimal current intensities necessary to elicit running and vocalization between wild-type and KO mice. KO mice showed a lower sensitivity to footshock, as assessed by jumping response. The current intensity used for fear conditioning (0.5 mA) was sufficient to generate a conditioned fear response in KO mice; thus, the lower sensitivity to footshock may not affect the fear conditioning (Figure 5a).

## Discussion

Short form Vesl is unique among the Vesl family proteins in that its expression is upregulated by various neural activities. Although Vesl-1 null mice have been generated [18,37], short-form-specific KO mice are lacking. In this study, we generated short-form-specific KO mice by replacing the genomic DNA corresponding to the 11 amino acids unique to Vesl-1S with a cDNA encoding C-terminal amino acids of Vesl-1L. Expression of the Vesl-1L protein in homozygous mice was similar to that of wild type mice in terms of brain region specificity and expression levels; thus, we conclude that the homozygous mice were indeed short-form-specific KO mice with normal Vesl-1 long form function.

Extensive behavioral analyses revealed a role for the short form of Vesl-1 in multiple processes of fear memory formation. The impairments observed cannot be attributed to indirect effects that are caused by the absence of short form throughout development, as homozygous KO mice showed phenotypes (other than LTM formation) that were similar to those of WT mice. Adult KO mice showed normal behavior in the open-field test, the light-dark test, and the elevated plus-maze test. Spine number and spine morphology were normal in adult KO mice. Electrophysiological analyses revealed that the basal synaptic transmission profile was normal (KN, HK, AMW, NI, KI, and TM, unpublished results). Furthermore, KO mice showed no apparent deficit in STM; therefore, absence of the short form of Vesl-1 appears to specifically affect LTM formation.

Knockout mice displayed deficits in LTM when trained with a mild (three footshocks) training paradigm. This LTM deficit, however, was rescued by trained with a stronger paradigm (five footshocks). This suggests that the short form of Vesl-1 plays a role in LTM formation when repeated reinforcement of the learning paradigm is lacking.

What is the molecular mechanism underlying the LTM deficit in KO mice? A strong correlation between LTM storage and hippocampal L-LTP has been indicated in several reports in which deficits in L-LTP in gene-manipulated mice are accompanied by impairment in LTM formation of hippocampus-dependent learning [38-40]. Activation of mGluR5 is necessary for L-LTP, via the phosphorylation of p70 S6 kinase, which is a major regulator of translation required for the protein synthesis-dependent L-LTP [41]. Vesl-1 proteins modulate the function of mGluRs. For example, the short and long forms of Vesl-1 regulate the neuritic targeting of mGluR5. Vesl-1S expression triggers the translocation of mGluR5 to both the dendrites and axons of cultured cerebellar granule cells [42]. Furthermore, binding to Vesl directly modulates the antagonist-independent activity of mGluRs [43], thus, the KO of the short form of Vesl-1 may lead to improper regulation of synaptic mGluR function, which in turn disturbs the establishment of L-LTP. Alternatively, the short form of Vesl-1 is involved in L-LTP through the regulation of ryanodine receptors (RyRs). Vesl-1L regulates calcium release from RyRs and Vesl-1S reverses the effects of Vesl-1L on RyR1 and RyR2 [15,16]. Because RyRs are involved in hippocampal L-LTP [44], dysfunction of RyR regulation in KO mice may lead to an impairment in L-LTP formation. This may result in a deficit in the consolidation of fear memory in KO mice.

This study further suggests that the short form of Vesl-1 is indispensable for the reconsolidation and within-session extinction of fear memory. mGluR5 is reported to be required for metaplasticity in hippocampal LTP [45]. Metaplasticity may be the physiological mechanism underlying memory reconsolidation. As mentioned above, Vesl-1S has the potential to regulate the function of mGluR5. We therefore suggest that disturbance of mGluR5 function in KO mice may affect metaplasticity, which in turn results in the defective reconsolidation and within-session extinction of fear memory.

## Conclusion

In this study, we successfully generated short form Vesl-1-specific KO mice without affecting the expression of the long form of Vesl-1, which is a splicing isoform of the *vesl-1 *gene. Analyses of KO mice revealed an important role of the short form of Vesl-1 in the multiple processes involved in the formation of long-term fear memory.

## Methods

### Animal experiments

All animal experiments were carried out in accordance with the National Institutes of Health guide for the care and use of laboratory animals and were approved by the Animal Care and Use Committee of the Mitsubishi Kagaku Institute of Life Science, MITILS.

### Targeting vector construct

A genomic DNA fragment from mouse 129 line including the *vesl-1 *gene region was cloned into the pBluescript II vector. The targeting construct was prepared using a DNA fragment containing a diphtheria toxin A fragment (DT-A) driven by the pgk promoter, a 1.5 kb NcoI-PstI fragment located in the 5' region of a *vesl-1S*-specific region in exon 5, a fragment containing exons 7–11 of the *vesl-1L *cDNA, a neo gene driven by the pgk promoter, an 11 kb KpnI-NotI fragment located 3' of the *vesl-1S*-specific region in exon 5, and the pBluescript plasmid (Figure 1c). This construct was designed to delete a *vesl-1S*-specific 394 bp fragment from the *vesl-1 *gene.

### Generation of *vesl-1 *short form-specific KO mice

Knockout mice were generated following standard procedures in which the targeting vector was transfected into 129/ola ES cells. Homologous recombinants were injected into C57BL/6 blastocysts to obtain chimeric mice. Male mice in which germline transmission had occurred were mated with C57BL/6 females to generate heterozygotes. Heterozygous animals were backcrossed to C57BL/6 mice 10 times to coordinate the genetic background, and the resulting animals were intercrossed to obtain homozygotes. All behavioral experiments were carried out using these mice.

### Northern blot analysis

Poly(A) RNA was isolated from whole brain using a commercial kit (Promega, Madison, WI). RNA was electrophoresed on a formaldehyde agarose gel. The gel was blotted onto an Immobilon-NY+ membrane (Millipore, Billerica, MA) and was then probed with full length *vesl-1L *fragment and reprobed with a fragment from G6PDH.

### Western blotting

Adult mice were subjected to MECS (100 Hz, 60 mA, 0.5 msec width, 0.1 sec). After 3 or 5 h, cortices were dissected and frozen in liquid nitrogen and were then homogenized in 0.3 ml of lysis buffer (5 mM HEPES pH 7.4, 5 mM EDTA pH 8.0, 1 mM Na_3_VO_4_, 10 mM NaF, 10 μg/ml leupeptin, 10 μg/ml aprotinin, 1 mM Pefablock, 714 ng/ml Pepstatin, 0.32 M sucrose). The homogenates were centrifuged at 1000 × g and the resulting supernatants were centrifuged at 17000 × g. Protein concentration was determined using the CBB method. SDS sample buffer was added and the homogenates were boiled for 10 min. The homogenates were electrophoresed on a 4–20% SDS-polyacrylamide gel (MULTIGEL II Mini 4/20, Daiichi Pure Chemicals, Tokyo)and blotted to an Immobilon-P membrane (Millipore, Billerica, MA). The membrane was blocked with TBST (40 mM Tris-HCl pH 7.4, 137 mM NaCl, and 0.05% Tween 20) containing 5% dried milk, and was then blotted with an anti-Vesl-1L antibody [12,27] and incubated with horseradish peroxidase (HRP)-conjugated secondary antibody. Signals were visualized using the enhanced chemiluminescence method (Supersignal West Femto Maximum sensitivity substrate, PIERCE, Rockford, IL).

### Immunohistochemistry

Adult mice were subjected to MECS (100 Hz, 45 mA, 0.2 msec width, 0.3 sec). After 5 h, brains were freshly frozen in powdered dry ice. Frozen sections (10 μm thick) were fixed with 4% paraformaldehyde for 30 min, washed with PBS, blocked in 5% BSA, and incubated in PBS containing a rabbit anti-Vesl-1L-specific antibody overnight. Sections were then incubated with an FITC-conjugated rabbit IgG overnight and were mounted in glycerol-based solution (MERCK, Darmstadt, Germany).

### Thionine staining

Adult mice were subjected to MECS (100 Hz, 45 mA, 0.2 msec width, 0.3 sec). After 3 h, brains were freshly frozen in powdered dry ice. Fresh frozen sections (10 μm thick) were fixed with 4% paraformaldehyde for 30 min, washed with PBS, and soaked in 0.1% thionine solution for 1 min. The samples were dehydrated serially from 70 to 100% ethanol, then 100% xylene, and were mounted in xylene-based mounting solution (MERCK, Darmstadt, Germany).

### Analyses of spine morphology using a neurotracer

We injected 0.08 μl of 10% solution of an anterograde neurotracer (biotinylated dextran amine, Molecular Probes, Carlsbad, CA) into the dorsal hippocampus of mice under nembutal anesthesia. Four days after the tracer injection, brains were fixed by cardiac perfusion with 4% paraformaldehyde and sectioned to 40 μm. Sections were incubated with HRP-conjugated streptavidin (Roche, Basel, Switzerland), followed by diaminobentidine (DAB) (Molecular probes, Carlsbad, CA) and Nickel treatment. Neurons of the hippocampus near the injection sites were sometimes necrotic. In addition, pyramidal neurons of the cortical layer II-III appeared healthy and were more sparsely labeled than those of the hippocampus. The tracer may have leaked out from the needle tip at the corpus callosum, which interconnects pyramidal neurons in the cortical layer II-III. We analyzed the spines on the first dendrite of pyramidal neurons, which is the dendrite between the soma and the first branching point in cortical layer II-III of the somatosensory and auditory cortices.

We counted the number of spines on the first arborization of the dendrite by looking through the ocular lens and serially turning the focus of each spine image (Figure 4g), which allowed us to count the number of spines more precisely when compared with counting using photo images. The length of the first dendrites was assessed using the Metamorph software (Molecular Devices, Downingtown, PA).

To assess spine length and spine head width, we scanned images sequentially at every 50 nm. The best focused image for each spine was used to measure the length and the width of spines. The length of individual spines was measured from the tip of the spine head to the interface with the dendritic stalk. The spine width was assessed at the widest part of the head with a line that was drawn perpendicularly to the line used for the spine length assessment. The edge of DAB staining was ambiguous; therefore, the illumination conditions of the room were kept constant, which allowed us to relatively identify similar edges of staining at every day of investigation.

### Contextual fear conditioning

Mice used for behavioral experiments were prepared using *in vitro *fertilization technique. Male mice were separated from females at birth and were nursed exclusively among males (maximum 10 males per cage) by foster female parents. Mice were kept on a 12 hr light/dark schedule. Mice of 11–18 weeks of age were used at the start of behavioral experiments. Mice were handled daily for 5 min for 5 consecutive days prior to behavioral experiments, to reduce stress. The apparatus and experimental conditions used for contextual fear conditioning were described previously [46]. Briefly, the dimensions of the apparatus were 16.5 cm × 17.5 cm × 30 cm, which contained 26 stainless steel rods from which electrofootshocks were delivered. Mice were placed into the chamber for 2 min, and the conditioned stimuli (the experimental chamber and context) were paired with aversive and unconditioned electric footshocks that consisted of the administration of 3, 5, or 8 times of 0.5 mA 0.5 sec shocks at 1 min intervals. Mice were then left for 3 min in the chamber. Conditioned freezing responses were automatically detected (NIH Image FZ2.15mr, OHARA IKA, Tokyo), which were characterized by an immobile and crouching posture observed upon subsequent presentation of the conditioned stimuli.

### Open-field test

General activity levels were measured using an open field. The apparatus and the conditions used were described previously [46]. The activity of animals placed in the open-field (50 × 50 cm) chamber were monitored for 30 min on 24 beams placed 2 cm above the floor and 48 beams placed 2 and 6 cm above the floor on another 2 faced side of the chamber. Locomotor distance and the number of rearings were automatically calculated (beam style small animal behavioral analyzing system version 2.0, MUROMACHI KIKAI, Tokyo).

### Light-dark test

The apparatus and conditioning used in this test were described previously [46]. The number of transitions from the light to the dark compartment and the stay time in the dark compartment were automatically calculated.

### Elevated plus-maze test

The apparatus used in this test consisted of a central platform (5 × 5 cm) and four arms (25 × 5 cm) placed 48 cm above the floor. Two arms were enclosed within translucent walls (15 cm high) and the other two arms (open) had low rims (1 cm high). Mice were placed in the center of the apparatus and their behavior was recorded for 10 min with a video camera located above the maze. The traveling distance and the stay time in the closed arms were automatically calculated.

### Electro sensitivity test

The general method used in this test was described previously [46]. After contextual fear conditioning test, mice were reentered into the conditioning chamber and were subjected to electrofootshocks ranging from weak electrical current to strong electrical current, in turn. The minimum electrical current value at which mice showed the first symptom of running, vocalization, or jumping was measured.

## Competing interests

The authors declare that they have no competing interests.

## Authors' contributions

NI designed and conducted all experiments, with the exception of the construction of the targeting vector, and contributed to the writing of the manuscript. MM, FH, TM, and KI conceived the original concept of this study. MM and FH designed and constructed the targeting vector. HN, NI, KN and AA generated the KO mice. KI supervised the entire project and wrote the manuscript.
